# Impact of Non-Persistence on Healthcare Resource Utilization and Costs in Patients With Immune-Mediated Rheumatic Diseases Initiating Subcutaneous TNF-Alpha Inhibitors: A Before-and-After Study

**DOI:** 10.3389/fphar.2021.752879

**Published:** 2021-11-29

**Authors:** Nuria Carballo, Enric Garcia-Alzórriz, Olivia Ferrández, María Eugenia Navarrete-Rouco, Xavier Durán-Jordà, Carolina Pérez-García, Jordi Monfort, Francesc Cots, Santiago Grau

**Affiliations:** ^1^ Pharmacy Department, Hospital del Mar—Parc de Salut Mar, Barcelona, Spain; ^2^ Universitat Autònoma de Barcelona, Barcelona, Spain; ^3^ Management Control Department, Hospital del Mar—Parc de Salut Mar, Barcelona, Spain; ^4^ Methodology and Biostatistics Support Unit, Institute Hospital del Mar for Medical Research (IMIM), Barcelona, Spain; ^5^ Department of Rheumatology, Hospital del Mar—Parc de Salut Mar, Barcelona, Spain

**Keywords:** rheumatic disease, persistence, biologics, healthcare resource consumption, rheumatoid arthritis, ankylosing spondylitis, psoriatic arthritis

## Abstract

Rheumatoid arthritis, psoriatic arthritis and ankylosing spondylitis are chronic progressive immune-mediated rheumatic diseases (IMRD) that can cause a progressive disability and joint deformation and thus can impact in healthcare resource utilization (HCRU) and costs. The main outcome of the study was to assess the effect of non-persistence to treatment with subcutaneous tumor necrosis factor-alpha inhibitors (SC-TNFis) on HCRU costs in naïve patients with IMRD who started treatment with adalimumab, etanercept, golimumab or certolizumab pegol during 12 months after initiation of treatment. The impact of persistence and non-persistence of SC-TNFis on HCRU costs was compared between 12 months before and 12 months after initiating SC-TNFis. Persistence was defined as the duration of time from initiation to discontinuation of therapy. The study was conducted in an acute care teaching hospital in Barcelona, Spain. Data for the period between 2015 and 2018 were extracted from the hospital cost management control database. HCRU costs comprised outpatient care, outpatient specialized rheumatology care, in-patient care, emergency care, laboratory testing and other non-biological therapies. The study population included 110 naïve SC-TNFis patients, divided into the cohorts of persistent (*n* = 85) and non-persistent (*n* = 25) patients. Fifty-six percent of patients were women, with a mean (standard deviation) age of 47.6 (14.8) years. Baseline clinical features and HCRU costs over the 12 months before the index prescription were similar in the two study groups. Before-and-after differences in mean (standard deviation) HCRU costs were significantly higher in the non-persistence group as compared to the persistence group for outpatient rheumatology care (€110.90 [234.56] vs. €20.80 [129.59], *p* = 0.023), laboratory testing (−€193.99 [195.88] vs. −€241.3 [217.88], *p* = 0.025), other non-biological drugs (€3849.03 [4046.14] vs. −€10.90 [157.42], *p* < 0.001) and total costs (€3268.90 [4821.55] vs. −€334.67 (905.44), *p* < 0.001). Treatment persistence with SC-TNFis may be associated with HCRU cost savings in naïve IMRD patients. Prescribing SC-TNFis with the best long-term persistence is beneficial.

## Introduction

Immune-mediated rheumatic diseases (IMRD) including rheumatoid arthritis (RA), psoriatic arthritis (PsA) and ankylosing spondylitis (AS) are chronic progressive diseases that impair and deteriorate joint function and structure (Sangha, 2000; Verstappen and Carmona, 2018). IMRD not only adversely affect patients in terms of diminished quality of life, pain, disability, or work-related productivity, but also represents a significant burden to society and healthcare systems due to high direct- and indirect-related healthcare costs (Li et al., 2006; Jacobs et al., 2011; Hsieh et al., 2020). The advent of biologics, particularly, subcutaneous tumor necrosis factor-alpha inhibitors (SC-TNFis) has marked a paradigm shift, revolutionizing the management of patients with IMRD (Kaneko and Takeuchi, 2014). However, taking the medication as prescribed and for a sufficient period of time is crucial for the success of treatment.

Poor adherence to long-term therapies severely, compromises effectiveness and safety outcomes, making patient adherence a critical issue from the perspective of the patient to achieve the lowest possible level of disease activity, optimize functional status and improve quality of life, as well as from the perspective of health economics (Chastek et al., 2017; Usherwood, 2017). Adherence to biological medication in RA has shown variability between 30 and 80%, mainly due to absence of a reference standard measure of adherence and differences in definition and terminology (Borah et al., 2009; Li et al., 2010). In the particular case of golimumab, a retrospective database analysis of 353 patients with rheumatic diseases receiving biological drugs, the probability of retention of golimumab at 1, 2, 3, 4, and 5 years was 85.9, 73.7, 68.5, 60.6, and 57.1%, respectively, with similar percentages across all indications and significantly greater when used as first biological agent compared with later therapies (Hernandez et al., 2019). In a systematic review of real-world treatment persistence of golimumab in the management of IMRD in Europe based on 27 studies, persistence at 24 months was about 50%, with lower persistence among axial AS (43%), but significantly better or equal persistence to other TNFis (Luttropp et al., 2019).

A number of studies have compared costs associated with persistence and non-persistence in patients with IMRD treated with SC-TNFis, including TNFi cycling, first-line and second-line TNFis prescription or switching to medications with a different mechanism of action (Dalén et al., 2016; Bhoi et al., 2017; Dalén et al., 2017; Bonafede et al., 2018; Lee et al., 2018; Sruamsiri et al., 2018; Ziegelbauer et al., 2018). Despite methodological differences, results of these studies provide evidence of non-persistence being more expensive, with higher costs to the healthcare systems of patients who discontinued their treatment compared to patients who were persistent.

All SC-TNFis, including adalimumab, etanercept, certolizumab pegol and golimumab are available in Spain and are approved for the treatment of RA, PsA and AS. A cost management control database established in our hospital provides an opportunity to evaluate costs associated with persistence versus non-persistence based on follow-up data of patients with IMRD treated with biological drugs in a real-world setting. The objective of the current study was to assess the impact of persistence compared with non-persistence to first-line SC-TNFis on healthcare resource utilization (HCRU) costs in IMRD.

## Materials and Methods

### Design and Patient Selection

This was a before-and-after observational cohort study conducted in a 400-bed acute-care teaching hospital in Barcelona, Spain. The primary objective of the study was to assess HCRU costs in naïve patients who initiated treatment with SC-TNFis for IMRD. Between January 1st, 2015, and December 30th, 2018, all consecutive patients aged 18 years or older diagnosed with RA (ICD-10 code M05.9), PsA (ICD-10 code L40.5), AS and other spondyloarthropathies (ICD-10 codes M08.1, M45, M48.8) who fulfilled the American College of Rheumatology/European League Against Rheumatism (ACR/EULAR) classification criteria, the classification criteria for Psoriatic arthritis (CASPAR), the Modified New York criteria (mNY) for AS or the Assessment of Spondylarthritis International Society (ASAS) classification criteria for axial SpA (axSpA) respectively, and who were candidates to initiate treatment with SC-TNFis were eligible. SC-TNFis naïve patients were defined as those who had never received a prescription of treatment with biologics before the observation period. Patients younger than 18 years of age, those who were lost to follow-up, and patients initiating treatment with intravenous TNFis and SC-TNFis for diseases other than IMRD were excluded from the study. The SC-TNFis of interest included adalimumab (Humira^®^), etanercept (Enbrel^®^), etanercept biosimilar (Benepali^®^, Erelzi^®^), golimumab (Simponi^®^) and certolizumab pegol (Cimzia^®^). The use of intravenous infliximab was excluded.

Time of initiation treatment with a SC-TNFis served as the index date and patients were followed up for 24 months (12 months prior to initiation treatment with SC-TNFis and 12 months after starting SC-TNFis). The treatment the year before starting TNFi was not evaluated, although according to current recommendations all patients had been treated with at least one conventional disease-modifying antirheumatic drugs (DMARDs).

The study protocol was approved by the Institutional Review Board and written informed consent was obtained from all patients at the time of prescribing SC-TNFis therapy. The present study was waived of informed consent as data of interest were collected from retrospective review of electronic medical records and administrative database.

### Data Source and Definitions

Data for this analysis was derived from a health administrative database of the hospital based on the GESCOT^®^ analytical accounting system. In this database, a unit cost is assigned for each activity, so that each act belongs to an episode and each episode to a patient. By grouping all the episodes of naïve patients who started therapy with SC-TNFis during the study period (2015–2018), the use of resources could be estimated. To calculate the HCRU costs, all acts whose registration date was included in the study period, including the previous 12 months, were captured. Data collection did not affect the treatment administered to patients.

Persistence was operationalized in accordance with the ISPOR Medication Compliance and Persistence Work Group definition (i.e., duration of time from initiation to discontinuation of therapy) (Cramer et al., 2008) and was estimated as the duration of time from SC-TNFis therapy initiation to discontinuation during 12 months of follow-up. Patients were considered non-persistent once a new prescription was not dispensed between the end of the last prescription and 60 days after the date of the last prescription (the grace period).

HCRU costs comprised outpatient care, outpatient specialized rheumatology care, in-patient care, emergency care, laboratory testing and other non-biological drugs (other non-biological therapies refer to drugs usually administered in subjects as outpatients but in the hospital setting, such as intravenous ferric carboxymaltose, zoledronic acid infusion or intravenous corticosteroids), and were estimated for the 12-months period before starting SC-TNFis and the following 12 months after the use of SC-TNFis. HCRU costs in the persistence and non-persistence cohorts were assessed. In addition, total healthcare costs were calculated as the mean of all allowable costs for medical claims and hospital pharmacy claims for each individual patient.

### Study Outcome

The outcome of the study was the effect of non-persistence to treatment with SC-TNFis on HCRU costs in naïve patients with IMRD during 12 months after initiation of therapy.

### Statistical Analysis

Data are expressed as frequencies and percentages for categorical variables and mean and standard deviation (SD) for quantitative variables. Variables between the non-persistence and persistence groups were compared with the chi-square (χ^2^) or the Fisher’s exact test for qualitative data and the Mann-Whitney *U* test for continuous data. The probability of retention of SC-TNFis treatment was assessed using Kaplan-Meier survival analysis and was expressed as the percentage of retention and the 95% confidence interval (CI). Differences according to RA, PsA and other IMRD were evaluated using the log-rank test. Statistical significance was at *p* < 0.05. Statistical analyses were performed using Stata version 15.1 for Windows.

## Results

At the time of data extraction, 110 patients (49 men and 61 women) with a mean (SD) age of 47.6 (14.8) years, fulfilled the inclusion criteria and were included in the study. They were divided into the groups of persistence and non-persistence, with 85 and 25 patients, respectively. The characteristics of these patients are shown in Table 1. There were no significant differences between the persistent and non-persistent groups in the distribution of patients according to IMRD disorder, with RA accounting for 43.6% of the cases followed by spondyloarthritis in 25.4%, and PsA in 11.8%. Forty-two patients (38.2%) were treated with etanercept, 29 (26.4%) with golimumab, 26 (23.6%) with adalimumab and 13 (11.8%) with certolizumab pegol.

**TABLE 1:** General characteristics of patients at initiation of SC-TNFis according to persistence and non-persistence with treatment at 12 months.

Variables	All patients (*n* = 110)	Persistence (*n* = 85)	Non-persistence (*n* = 25)	*p* value
Gender				0.493
Male	49 (44.5)	36 (42.3)	13 (52.0)	
Female	61 (55.4)	49 (57.6)	12 (48.0)	
Age, years, mean (SD)	47.6 (14.8)	47.3 (15.4)	48.6 (12.7)	0.692
Race				0.351
Caucasian	97 (88.2)	75 (88.2)	22 (88.0)	
Asiatic	8 (7.2)	5 (5.9)	3 (12.0)	
Other	5 (4.5)	5 (5.9)	0 (0.0)	
IMRD				0.470
Rheumatoid arthritis	48 (43.6)	34 (40.0)	14 (56.0)	
Psoriatic arthritis	13 (11.8)	11 (12.9)	2 (8.0)	
Ankylosing spondylitis	28 (25.4)	24 (28.2)	4 (16.0)	
Other spondyloarthropathies	21 (19.1)	16 (18.8)	5 (20.0)	
Treatment with SC-TNFis				0.398
Etanercept	42 (38.2)	28 (32.9)	14 (56.0)	
Etanercept biosimilar	27 (24.5)	17 (20.0)	10 (40.0)	
Golimumab	29 (26.4)	24 (28.2)	5 (20.0)	
Adalimumab	26 (23.6)	22 (25.9)	4 (16.0)	
Certolizumab pegol	13 (11.8)	11 (12.9)	2 (8.0)	

Data expressed as frequencies and percentages in parenthesis unless otherwise stated; SD: standard deviation; IMRD: immune-mediated rheumatic disease; SC-TNFis: subcutaneous tumor necrosis-alpha inhibitors.

**FIGURE 1:**
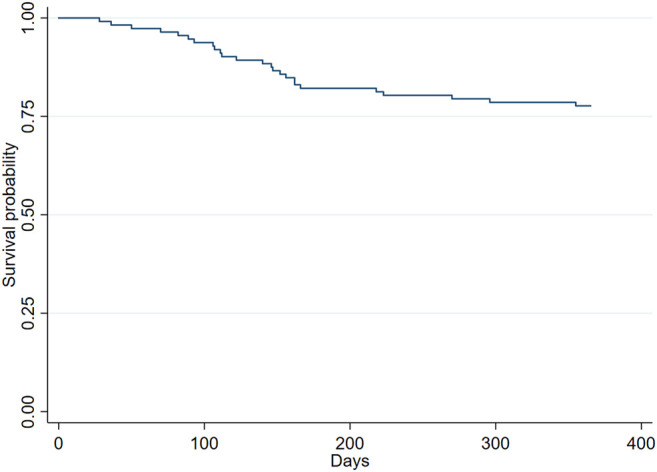
Overall rate of retention of treatment with SC-TNFis at 12 months after initiation of treatment in naïve patients with IMRD.

**FIGURE 2:**
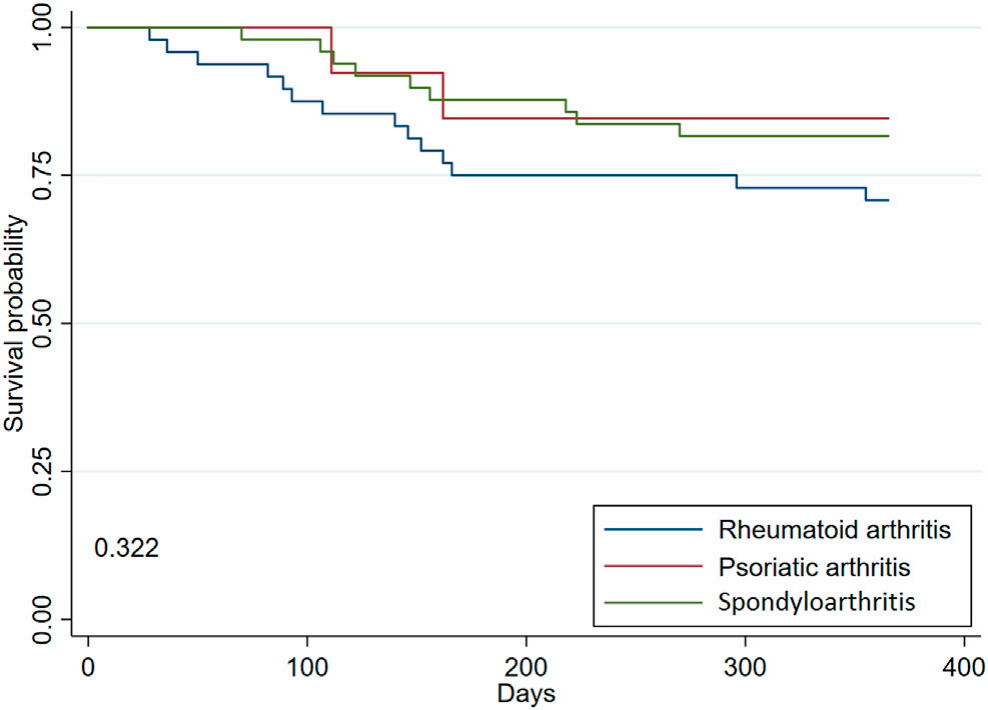
Rate of retention of treatment with SC-TNFis at 12 months after initiation of treatment in naïve patients with IMRD according to the underlying rheumatic disease.

In the Kaplan-Meier analysis, the overall rate of retention of SC-TNFis was 77.3% (95% CI 68.8–84.3) (Figure 1). As shown in Figure 2, the retention rates of SC-TNFis were 84.6% (95% CI 51.2–95.9) for PsA, 81.6% (95% CI 67.7%–90%) for spondylarthritis including AS and other spondyloarthropathies, and 70.8% (95% CI 55.8%–81.6%) for RA (log-rank 0.322). Reasons of non-persistence were lack of efficacy in 16 (64%) patients, adverse events in 7 (28%), and poor adherence in 2 (8%). Reported adverse events were injection site reactions, upper respiratory tract infection, hepatotoxicity, adverse cutaneous reactions, and flu-like syndrome.

Details of HCRU costs before and after initiation of treatment with SC-TNFis are shown in Table 2. There were statistically significant differences in the overall costs, costs associated with outpatient specialized rheumatology care, and costs of other non-biological drugs in favor of the persistence group. Mean (SD) differences in HCRU costs after and before the 12-months study periods for patients in the persistent vs. non-persistent groups were €20.80 (129.59) and €110.90 (234.56) (*p* = 0.023) for outpatient rheumatology care, −€241.3 (217.88) and −€193.99 (195.88) (*p* = 0.025) for laboratory testing, −€10.90 (157.42) and €3849.03 (4046.14) (*p* < 0.001) for treatment with other non-biological drugs, and −€334.67 (905.44) and €3268.90 (4821.55) (*p* < 0.001) for total costs.

**TABLE 2:** Healthcare resource utilization costs in the group of persistent and non-persistence patients with SC-TNFis.

Variables	Total (*n* = 110)	Persistence (*n* = 85)	Non-persistence (*n* = 25)	*p* value
12 months before SC-TNFis				
Overall costs	1007.59 (1402.87)	896.60 (1247.60)	1384.94 (1816.17)	0.299
Outpatient care	122.70 (471.20)	87.17 (293.61)	243.48 (828.86)	0.204
Outpatient rheumatology care	184.24 (120.55)	174.79 (1247.60)	216.39 (169.88)	0.224
In-patient care	245.36 (120.55)	170.34 (846.47)	500.41 (1542.93)	0.571
Emergency care	38.95 (79.31)	39.30 (83.16)	37.77 (66.0)	0.850
Laboratory testing	385.46 (203.70)	388.20 (207.07)	376.12 (195.59)	0.458
Other non-biological therapies	30.88 (221.01)	36.79 (250.55)	10.77 (39.83)	0.803
12 months after SC-TNFis				
Overall costs	1491.91 (2709.23)	561.93 (682.14)	4653.84 (4269.61)	<0.001
Outpatient care	83.36 (128.67)	76.67 (112.90)	106.11 (172.85)	0.682
Outpatient rheumatology care	225.52 (130.99)	195.58 (100.05)	327.29 (170.10)	<0.001
In-patient care	82.79 (460.11)	80.86 (466.54)	89.35 (446.77)	0.969
Emergency care	48.12 (125.31)	36.06 (106.23)	84.14 (171.89)	0.198
Laboratory testing	154.88 (138.89)	146.86 (141.48)	182.14 (128.62)	0.061
Other non-biological therapies	897.24 (2493.21)	25.89 (116.05)	3859.80 (4043.86)	<0.001
After vs. before SC-TNFis difference				
Overall costs	484.32 (2837.59)	−334.67 (905.44)	3268.90 (4821.55)	<0.001
Outpatient care	−39.33 (477.60)	−10.50 (305.0)	−137.37 (835.11)	0.735
Outpatient rheumatology care	41.28 (162.77)	20.80 (129.59)	110.90 (234.56)	0.023
In-patient care	−162.57 (942.33)	−89.48 (603.86)	−411.05 (1635.05)	0.610
Emergency care	9.17 (133.83)	−3.25 (113.38)	51.38 (184.26)	0.473
Laboratory testing	−230.58 (213.14)	−241.3 (217.88)	−193.99 (195.88)	0.025
Other non-biological therapies	866.36 (2502.87)	−10.90 (157.42)	3849.03 (4046.14)	<0.001

Data expressed as mean and standard deviation in €; SC-TNFis: subcutaneous tumor necrosis factor-alpha inhibitors. Overall costs included outpatient care, outpatient specialized rheumatology care, in-patient care, emergency care, laboratory tests, and other non-biological therapies. Other non-biological therapies refer to drugs usually administered in subjects as outpatients but in the hospital setting, such as intravenous ferric carboxymaltose, zoledronic acid infusion or intravenous corticosteroids.

## Discussion

In the treatment of IMRD, biologic agents are recommended for patients who have experienced an inadequate response to conventional DMARDs. Among the biologics currently approved for treatment of naïve patients with IMRD, we have selected adalimumab, etanercept, golimumab and certolizumab pegol that can be administered through subcutaneous injection as these TNFis are usually the first-line options when initiating a patient with IMRD on biologic therapy (Williams and Edwards, 2006; Chilton and Collett, 2008). This single-center before-and-after study of patients with IMRD starting treatment with SC-TNFis for the first time shows that persistence with SC-TNFis treatment using a 12-months period framework in comparison with the non-persistent cohort was associated with significant reductions in total HCRU costs as well as costs of outpatient rheumatology visits, laboratory testing, and the use of other non-biological drugs. The rate of non-persistence of 22.7% (25/110) found in our study is similar to data reported in other studies. In a total of 1,005 patients with AS treated with TNFis collected from the Korean College of Rheumatology Biologics Registry, discontinuation of TNFis occurred in 24.2% after a median follow-up of 14 months (Kim et al., 2021). In a study that reported drug retention rates in 24,915 biologic-naïve patients with axial SpA initiating TNFis treatment from 12 registries in the EuroSpa collaboration, the 12-months non-retention rate was 20% (Ørnbjerg et al., 2019). In addition, persistence rates can vary notably according to the country, characteristics of the healthcare services, or type of specific drug being evaluated. In our study, however, the comparison of the persistent and non-persistent cohorts before initiation of SC-TNFis therapy did not reveal significant differences, leading us to believe that persistence may not be associated with SC-TNFis costs offsets in patients with IMRD.

Other studies have also shown the positive impact of persistence on costs in patients with different rheumatic diseases treated with SC-TNFis. In a retrospective cohort study based on the German statutory health insurance database and using a 2-years time horizon, after 1:1 matching 678 persistent and 678 non-persistent patients, the cost of office-based visits, hospitalizations, co-medications, and sick leave were higher in patients who discontinued SC-TNFis after an average of 9 months compared with patients with at least 24 months of therapy (Ziegelbauer et al., 2018). In a study in which data were extracted from a Japanese claims database, of a total of 6,153 naïve patients treated for the first time with biological DMARDs for RA, the non-persistent group had a larger increase in outpatient visits, with persistence associated with a reduction of total healthcare costs of US$760 (Sruamsiri et al., 2018). The reduction in medication costs in non-persistent patients was offset by higher hospitalization costs, making non-persistence more expensive (Sruamsiri et al., 2018). In a large study population of 4,903 patients treated for the first time with SC-TNFis and 845 with their second SC-TNFi identified from the Swedish Prescribed Drug Register between May 2010 and December 2012, patients treated with the second SC-TNFi had significantly lower persistence and incurred in higher costs (Svedbom et al., 2017).

As far as we are aware, there is only one study that included a cost analysis of HCRU costs prior to initiation of SC-TNFis and 12 months post-initiation in 1,793 persistent and 1,326 non-persistent patients (Dalén et al., 2016), which is similar to our before-and-after design. They found significant differences in HCRU costs post- and prior initiation in specialized outpatient care, in-patient care, non-DMARD medication in the group of persistent patients, and in costs of in-patient care only in the group of non-persistence (Dalén et al., 2016). Although in this study between-group comparisons between the persistent and non-persistent groups are not presented, differences in the persistent group were higher than in the non-persistent group, which is consistent with results of our study. Other studies have shown higher persistence and substantially lower cost per persistent patient for those who switched from a TNFi to another drug with an alternative mechanism of action, rather than in patients who cycled between TNFis (Bonafede et al., 2018). This aspect, however, was not analyzed in the present study as only newly treated patients with SC-TNFis were included. On the other hand, costs of biologic drugs per effectively treated patient according to claims-based algorithm have been reported, with etanercept having the lowest cost (Curtis et al., 2015).

The present findings, however, should be interpreted considering the limitations of the study including the single-center design, the small number of patients included in the non-persistent group and the fact that differences in HCRU costs stratified by biologic drugs were not determined. Moreover, the total number of patients treated with different SC-TNFis for different diseases (RA, PsA, AS and other spondyloarthropathies) is relatively small. Also, as this study was conducted on a hospital-based administrative management database, data of which were integrated in the healthcare system of Catalonia, Spain, the results could not be generalizable to other settings. However, the present findings have value in daily clinical practice. The present results were evaluated collectively, and it would be interesting to analyze HCRU separately and to compare HCRU for the different disorders in further studies. The causes of non-persistence were not assessed, although lack of efficacy was the main reason (64% of patients) followed by adverse events and poor adherence.

In conclusion, in patients with IMRD initiating first-line SC-TNFis, persistence in treatment over a study period of 12 months was associated with decreased HCRU costs in outpatient rheumatological care, laboratory services, use of other non-biological therapies and total costs. Considering the lower costs for persistent patients, adherence to SC-TNFis continues to be a crucial factor given its impact on patients and payers.

## Data Availability

The raw data supporting the conclusion of this article will be made available by the authors, without undue reservation.
