# Corrigendum: Impact of Non-Persistence on Healthcare Resource Utilization Costs in Patients With Immune-Mediated Rheumatic Diseases Initiating Subcutaneous TNF-Alpha Inhibitors: A Before-and-After Study

**DOI:** 10.3389/fphar.2022.867017

**Published:** 2022-03-07

**Authors:** Nuria Carballo, Enric Garcia-Alzórriz, Olivia Ferrández, María Eugenia Navarrete-Rouco, Xavier Durán-Jordà, Carolina Pérez-García, Jordi Monfort, Francesc Cots, Santiago Grau

**Affiliations:** ^1^ Pharmacy Department, Hospital del Mar – Parc de Salut Mar, Barcelona, Spain; ^2^ Universitat Autònoma de Barcelona, Barcelona, Spain; ^3^ Management Control Department, Hospital del Mar – Parc de Salut Mar, Barcelona, Spain; ^4^ Methodology and Biostatistics Support Unit, Institute Hospital del Mar for Medical Research (IMIM), Barcelona, Spain; ^5^ Department of Rheumatology, Hospital del Mar – Parc de Salut Mar, Barcelona, Spain

**Keywords:** rheumatic disease, biologics, persistence, healthcare resource utilization and costs, anti TNF agents

In the published article, there was an error regarding the affiliations for **Nuria Carballo, Olivia Ferrández, Jordi Monfort and Santiago Grau**. These authors all have an additional affiliation, listed as “**Universitat Autònoma de Barcelona, Barcelona, Spain**”.

In addition, there was an error in the Acknowledgements section in the original article. The following statement was should have been included:

“This study is part of a PhD programme in Medicine of the Universitat Autònoma de Barcelona. This study was presented, in part, as a poster at the EULAR Virtual Congress, 2–5 June 2021 in Paris, France.”

The authors apologize for these errors and state that they do not change the scientific conclusions of the article in any way.

